# MicroRNAs in spermatogenesis dysfunction and male infertility: clinical phenotypes, mechanisms and potential diagnostic biomarkers

**DOI:** 10.3389/fendo.2024.1293368

**Published:** 2024-02-16

**Authors:** Ziyan Shi, Miao Yu, Tingchao Guo, Yu Sui, Zhiying Tian, Xiang Ni, Xinren Chen, Miao Jiang, Jingyi Jiang, Yongping Lu, Meina Lin

**Affiliations:** ^1^ NHC Key Laboratory of Reproductive Health and Medical Genetics & Liaoning Key Laboratory of Reproductive Health, Liaoning Research Institute of Family Planning, China Medical University, Shenyang, China; ^2^ Department of Biochemistry & Molecular Biology, China Medical University, Shenyang, China; ^3^ Science Experiment Center, China Medical University, Shenyang, China

**Keywords:** male infertility, sperm, MicroRNAs, NOA, diagnostic markers

## Abstract

Infertility affects approximately 10–15% of couples worldwide who are attempting to conceive, with male infertility accounting for 50% of infertility cases. Male infertility is related to various factors such as hormone imbalance, urogenital diseases, environmental factors, and genetic factors. Owing to its relationship with genetic factors, male infertility cannot be diagnosed through routine examination in most cases, and is clinically called ‘idiopathic male infertility.’ Recent studies have provided evidence that microRNAs (miRNAs) are expressed in a cell-or stage-specific manner during spermatogenesis. This review focuses on the role of miRNAs in male infertility and spermatogenesis. Data were collected from published studies that investigated the effects of miRNAs on spermatogenesis, sperm quality and quantity, fertilization, embryo development, and assisted reproductive technology (ART) outcomes. Based on the findings of these studies, we summarize the targets of miRNAs and the resulting functional effects that occur due to changes in miRNA expression at various stages of spermatogenesis, including undifferentiated and differentiating spermatogonia, spermatocytes, spermatids, and Sertoli cells (SCs). In addition, we discuss potential markers for diagnosing male infertility and predicting the varicocele grade, surgical outcomes, ART outcomes, and sperm retrieval rates in patients with non-obstructive azoospermia (NOA).

## Graphical Abstract

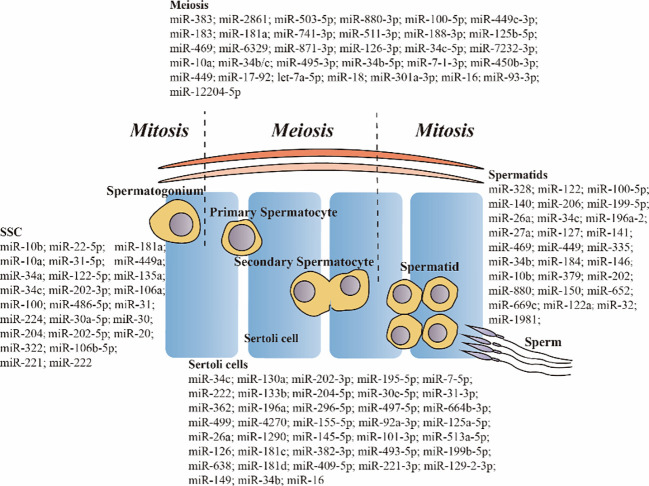

## 1 Introduction

The World Health Organization (WHO) defines infertility as 12 months of unprotected intercourse without a successful pregnancy. Infertility is a global reproductive health problem that affects fertility and population growth rates. This is an objective factor affecting birth rates. Approximately 10–15% of couples experience infertility worldwide, with male infertility accounting for 50% of cases. The incidence of infertility varies greatly among countries and regions. The incidence of male infertility is highest in Central and Eastern Europe (8–12%) and lowest in Sub-Saharan Africa (2.5–4.8%) (1). The WHO revised its guidelines for semen analysis for the diagnosis of male infertility in 2010 and 2015, resulting in significantly lower reference values and more men qualifying as ‘normal.’ Male reproductive problems are major health concerns worldwide.

Male infertility is a complex and multifactorial pathological condition with highly heterogeneous phenotypic presentations, including azoospermia, oligozoospermia, oligoasthenospermia, teratozoospermia and cryptorchidism. It is related to various factors such as hormones, urogenital diseases, environmental factors, and genetic factors. Approximately 15–25% of male infertility cases are attributed to genetic factors. Particularly, patients with azoospermia have the highest risk of genetic anomalies (2).

Male infertility cannot be diagnosed through routine physical examination, semen analysis, or blood tests in approximately 60–75% of cases (3). This condition is clinically called ‘idiopathic male infertility,’ with non-obstructive azoospermia (NOA) being the most serious and difficult to treat. NOA accounts for approximately 10% of male infertility and 60% of azoospermia cases. It is characterized the absence of sperm cells in the ejaculate owing to inactive spermatogenesis. Currently, the clinical diagnosis of NOA depends on testicular biopsy and treatment depends on sperm retrieval through microdissection testicular sperm extraction (micro-TESE) and *in vitro* fertilization using intracytoplasmic sperm injection (ICSI). However, testicular biopsy may lead to postoperative complications such as tissue adhesion and testicular failure. Moreover, the success rate of sperm retrieval is limited. Therefore, it is necessary to develop a precise, non-invasive diagnostic method to classify the causes of sperm defects and predict the spermatogenic capacity of the testis. The use of non-invasive diagnostic methods may play a major role in overcoming the challenges associated with male infertility (3). Owing to their significant stability and relative ease of detection, microRNAs (miRNAs) may represent ideal tools for the rapid and noninvasive diagnosis of numerous diseases (4).

miRNAs are short noncoding RNAs (22–24 nucleotides in length) that regulate gene expression by binding to the 3´-untranslated regions (3´-UTRs) of many protein-coding transcripts. Each miRNA has several target mRNAs that possess multiple binding sites for the same or different miRNAs. miRNAs play a crucial role in different biological processes including differentiation, apoptosis, cell programming, tissue formation, embryonic development, organogenesis and growth. Given that miRNAs exhibit tissue-specific and developmentally regulated expression patterns, miRNA disorder is thought to be directly related to the pathogenesis of various diseases, including developmental disorders, cancer and infertility. Several recent studies have demonstrated that miRNAs play a pivotal role in spermatogenesis, oocyte fertilization and somatic cell development. In addition, aberrant expression of certain miRNAs is associated with male infertility with different testicular patterns (such as Sertoli cell-only and germ cell arrest), spermatogenic impairment (such as azoospermia and oligospermia) and azoospermia. Therefore, miRNAs are promising biomarkers for the diagnosis of male infertility and assessment of spermatogenesis status (5).

## 2 miRNAs and abnormal sperm quality

High-quality spermatozoa exhibit properties conducive to fertilization such as normal morphology, good motility, and the absence of DNA fragmentation. However, low-quality spermatozoa have morphological defects, low motility, and a high degree of DNA fragmentation, all of which may result in azoospermia, oligoasthenoteratozoospermia and teratozoospermia (6). Azoospermia is characterized by absent or low sperm motility, defined as a decrease in total motility (<40%) and progressive motility (<32%) in semen samples (7). oligoasthenoteratozoospermia is characterized by a decreased sperm concentration accompanied by significant abnormalities in sperm morphology and motility. Based on the WHO criteria (5th edition, 2010), teratozoospermia is characterized by abnormalities in the morphological characteristics of sperm and the presence of <4% normal spermatozoa in the ejaculate. Poor sperm quality negatively affects fertilization and embryonic development. Several miRNAs have been associated with abnormal sperm quality (**Table 1**).

**Table 1:** miRNAs related to abnormal sperm quality.

Ref	miRNA	Studied patients	Position	miRNA measure	Comparison	Variation	Target genes	Effects on spermatogenesis
(8)	Let-7a; let-7d; let-7e; miR-22	AB=7; LM=8; N=7	S	qRT-PCR;	AB vs N	up	*Hmg2*	NA
	miR-15b					down		
	let-7d; let-7e				LM vs N	down		
(9)	miR-122; miR-34b; miR-34c-5p; miR-16	AZS=9; OAZ=9; N=9;	S	Microarray; qRT-PCR	OAZ vs N	down	NA	NA
	miR-141; miR-200a;					up		
	miR-122; miR-34b				AZS vs N	down		
	miR-141; miR-200a					up		
(10)	miR-34-b/c; miR-449	DKO=8; WT=17	T	qRT-PCR; RNA-Seq	DKO vs WT	down	NA	Positively correlated with sperm quantity, morphology
(11)	miR-34-b/c; miR-449	DKO=12; WT=8	T	Microarray; qRT-PCR	DKO vs WT	down	NA	Positively correlated with sperm quality and morphology
(12)	miR-27b	AZS=24; N=24	S	qRT-PCR;	AZS vs N	up	*Crisp2*	Influence sperm morphology and progressive motility.
(13)	miR-34b-3p;	AZS=10; TZ=10; O=10	S	qRT-PCR; Small-RNA Chips; Nano-RNA chips	NA	NA	NA	Positively correlated with age
	miR-629-3p				AZS vs N			Negatively correlated with sperm motility
	miR-335-5p; miR-885-5p; miR-152-3p				O vs N			Negatively correlated with sperm concentration values
	miR-2861					down		
(14)	miR-30b; miR-15a	OAZ=12; N=12	SP	Microarray; qRT-PCR	OAZ vs N	down	*NA*	NA
	miR765; miR-1299; miR-1275					up		
(15)	miR-541	MC-LR=15; N=5	T	qRT-PCR	MC-LR vs N	up	*p15*	Promotes cell viability and reduces cell apoptosis.
(16)	miR-34a	NA	T	qRT-PCR	KO vs N	down	*gsk3a*	Negatively correlation with sperm motility parameters
(17)	miR-26a-5p; let-7g-5p	HC=15; N=15	S/Sp	qRT-PCR; RNA-Seq	NI vs NF	down	*Pten;* *Pmaip1*	Positively correlation with sperm quality
(18)	miR-888-3p	AZS=39; N=35	S	Sequencing; qRT-PCR	AZS vs N	up	NA	NA
(19)	miR-146a; miR-371; miR-122	elderly=40 Young=40	SP	TaqMan Array; qRT-PCR	Elderly vs young	down	NA	NA
(20)	miR-138	NA	GC2 cells	microRNA array; qRT-PCR	SiNP vs N	up	*Fas;Fasl* *Ripk1;* *Fadd*	Regulating GC2 apoptosis.
(21)	miR-125b-2	KO=8; WT=8	S	qRT-PCR	KO vs WT	down	*Pap*	NA
(22)	miR-200a-5p; miR-141-3p	HA	S	qRT-PCR	bad vs good breeders	up	*Dmrt1;* *Kita*	NA
	miR-122-5p					down		
(23)	miR-106a	infertile =111	Sp	qRT-PCR	NA	down	NA	Positively correlated with sperm motility.
(24)	miR-26a-5p	NI=15; NF=15	S	qRT-PCR	NI vs NF	down	*Pten*	Positively correlated with sperm motility and normal morphology.
(25)	miR-34a, b, c	AZS=22; TZ=25; N=52 ATS=28; OAT=27;	S	qRT-PCR MS-PCR	AZS vs N TZ vs N ATS vs N OAT vs N	down	NA	Positively correlated with sperm count, progressive motility, and normal morphology.
(26)	miR-182-5p; miR-192-5p; miR-493-5p	TZ=21; N=20	S	qRT-PCR;	TZ vs N	up	*Crisp3*	NA
(27)	miR-191	IVF=102	S	RNA-Seq	High vs low	down	NA	Maintaining normal sperm morphology.
(28)	miR-574	NA	S/SP	RNA-seq; qRT-PCR	Aging vs Young	up	*ND5*	regulating mitochondrial function and ATP generation
(29)	miR-let-7a; miR-let-7b; miR-let-7c;	NOA=20; OAT=46; N=50	Sp	qRT-PCR	NOA vs N	up	NA	NA
	miR-518f					down		
(30)	let7a-5p; miR-9-3p; miR-22-5p; miR-30b-5p; miR-103-3p; miR-122-5p; miR-335-5p	OS=41; NI=40; AZS=40; NF=40	S	Microarray; qRT-PCR;	NI vs NF OS/AZS vs NF	down	NA	Positively correlated with sperm motility.
(31)	miR-10a-5p; miR-15b-5p; miR-26a-5p; miR-34b-3p; miR-122-5p; miR-125b-5p; miR-191-5p; miR-296-5p; let-7a-5p	TZ=13; N=15	S	qRT-PCR	TZ vs N	down	NA	positively correlated with each other and with spermatozoa morphology.
(32)	miR-143-3p	HS=14; N=6	T	qRT-PCR	HS vs N	up	*Bcl2*	Positively correlate with sperm motility
(33)	miR-450b-3p	SiNP=40; N=40	T	qRT-PCR	SiNP vs N	down	*Layilin* *Talin;* *Vinculin*	Regulating GC2 apoptosis.

AZS, asthenozoospermia; ATS, asthenoteratozoospermia; OAZ, Oligoasthenozoospermia; N, normal spermatogenesis; NF, normozoospermic fertile; NI, normozoopspermia infertile; S, Sperm; SP, seminal plasm: T, Testis; OAT, Oligoasthenoteratozoospermia; TZ, teratozoospermia; HC, hemicastration; AB, abnormal morphology; LM, sperm with morphological abnormalities, whereas low motility samples; FR, fertilization rate; HQER, high-quality embryo rate; EER, effective embryo rate; HS:heat stress; PAHs, polycyclic aromatic hydrocarbons; BPA, Bisphenol A; MS-PCR, methylation-specific PCR.

The miR-34/449 family plays a crucial role in normal testicular function, successful spermatogenesis, and the regulation of spermatozoa maturation and function. Abnormal expression of miR-34/449 family members contributes to the development of oligoasthenoteratozoospermia and infertility. In particular, miR-34b/c and miR-449 are highly expressed in postmitotic male germ cells. Deficiency of miR-34bc/449 disrupts both meiosis and the final stages of sperm maturation. miR-34a is ubiquitously and weakly expressed in normal tissues during spermatogenesis. In a previous study, zebrafish with miR-34a knockout (KO) exhibited normal testis morphology and sperm quantity and an increase in progressive sperm motility. In addition, miR-34a-KO male zebrafish had higher fertilization rates than wild-type (WT) male zebrafish (16). In another study, the expression levels of miR-34a, miR-34b, and miR-34c were examined in sperm samples collected from 102 infertile (azoospermia, teratozoospermia, asthenoteratozoospermia, and oligoasthenoteratozoospermia) and 52 fertile men (25). The expression of these three miRNAs was significantly lower in sperm samples of infertile men than in those of fertile men, with the expression of miR-34c-5p being the lowest in patients with oligoasthenoteratozoospermia. Additionally, the frequency of methylation in the promoter region of miR-34b/c was higher in infertile men than that in fertile men (82.4% and 23.3%, respectively). The highest frequencies of methylation were observed in patients with asthenoteratozoospermia (92.9%) and oligoasthenoteratozoospermia (93.8%). Men with low miR-34c-5p expression exhibited low sperm motility and normal sperm morphology (34).

Abu-Halima et al. analyzed 1,205 the expression profiles of in men with OA and fertile men with normozoospermia using a microarray platform. Seven miRNAs were upregulated, and 29 miRNAs were downregulated in men with OA. The quantitative reverse transcription-polymerase chain reaction (qRT-PCR) results validated that men with OA had significantly higher levels of miR-765/-1275 and lower levels of miR-15a (14). In addition, miR-122, -141 and -200 showed the highest fold changes in men with azoospermia and OA. miR-141-3p and miR-200a-5p have been proposed as markers of sperm motility in zebrafish. miR-141-3p and miR-200a-5p were upregulated, whereas miR-122-5p was downregulated in poor zebrafish breeders. Fertilization with low-quality sperm may lead to permanent molecular alterations in the progeny (22). Abu-Halima, et al (9) analyzed the miRNA expression profiles of nine men with normozoospermia, nine men with azoospermia, and nine men with OA using a microarray platform and RT-PCR. Compared to men with normozoospermia, men with azoospermia had 50 upregulated and 27 downregulated messenger RNAs (mRNAs), whereas men with OA had 42 upregulated and 44 downregulated miRNAs. miR-34b/-122/-1973 exhibited the highest fold change in men with azoospermia, and the expression of miR-34b/-34b*/-15b/-34c-5p/-122/-449a/-1973/-16/-19a was significantly altered in men with OA. In addition, the expression of miR-141/-200a/-122/-34b/-34c-5p/-16 was validated using qRT-PCR (9). Another study demonstrated that the expression of miR-182-5p/-192-5p/-493-5p was higher in men with teratozoospermia, with good area under the curve (AUC) values, suggesting that these miRNAs can be used as biomarkers for teratozoospermia (26).

Another study demonstrated that the expression of miR-182-5p/-192-5p/-493-5p was higher in men with teratozoospermia, with good area under the curve (AUC) values, suggesting that these miRNAs can be used as biomarkers for teratozoospermia (35). miR-27b is upregulated in ejaculated spermatozoa from patients with azoospermia, and its high expression is associated with low sperm progressive motility and is negatively associated with the levels of CRISP2 protein (12). The expression of hsa-miR-9-3p/-30b-5p/-103-3p/-122-5p was lower in men with oligospermia/oligoasthenozoospermia and azoospermia than in fertile men with normozoospermia, whereas that of hsa-let-7a-5p was lower in men with azoospermia. The expression of hsa-miR-9-3p/-30b-5p/-122-5p is positively associated with sperm concentration, whereas the expression of hsa-let-7a-5p and hsa-miR-9-3p/-30b-5p/-103-3p/-122-5p is positively associated with sperm motility (30). The expression of hsa-miR-9-3p/-30b-5p/-103-3p/-122-5p was lower in men with oligospermia/oligoasthenozoospermia and azoospermia than in fertile men with normozoospermia, whereas that of hsa-let-7a-5p was lower in men with azoospermia (21).

The expression of nine miRNAs, namely miR-10a-5p, miR-15b-5p, miR-26a-5p, miR-34b-3p, miR-122-5p, miR-125b-5p, miR-191-5p, miR-296-5p, and miR-let-7a-5p, was lower in the spermatozoa of patients with teratozoospermia than in those of control individuals. These miRNAs are positively associated with each other, and their abundance is positively associated with spermatozoa morphology (31). A previous study reported a significant association between the expression of hsa-mir-191-5p and sperm morphology, but no significant association was observed between sperm density and viability (27). Galehdari et al. (24) demonstrated that high expression of miR-26a-5p in sperm is associated with high sperm motility and normal sperm morphology. Overexpression of miR-26a-5p and let-7g-5p in freshly collected pig semen inhibited sperm apoptosis and improved sperm motility (17). Paoli et al. (19) showed that semen quality decreases with age, leading to a decrease in semen volume, progressive motility, total sperm number, number of progressively motile sperm, and an increase in the proportion of abnormal sperm.

This age-dependent decline was associated with the low expression of miR-122/-371/-146a in the seminal plasma of elderly men. A previous study demonstrated that the expression of miR-let-7d/-7e was significantly higher in pigs with low sperm motility than in control pigs (8). miR‐888‐3p has been reported to be significantly overexpressed in azoospermia (18), and the expression of miR-629-3p has been associated with sperm motility (13). The expression of miR-182-5p/-192-5p/-493-5p is higher in infertile men with teratozoospermia, whereas that of the CRISP3 glycosylated isoform, the target gene of the three miRNAs, is significantly lower in men with teratozoospermia than in healthy men, and is positively associated with normal sperm morphology in patients with teratozoospermia (26).

Mitochondria-related miR-574 is increased in the sperm of aging men and is inversely associated with sperm motility, particularly progressive motility; however, no significant association was observed between miR-574 expression and sperm concentration. This phenomenon may occur due to the inhibitory effect of miR-574 on mitochondrial function, which results in decreased cellular ATP production by directly targeting mt-ND5 (28).

Spermatogenesis is influenced by temperature, and an elevated in testicular temperature has a significant effect on mammalian spermatogenesis and semen quality. High-throughput sequencing analysis showed that 175 miRNAs were altered after heat stress (mice were placed in a 43°C water bath for 25 min). Among the altered miRNAs, miR-143-3p has been identified as a key regulatory factor that affects spermatogenesis under heat stress (32).

Silica nanoparticles (SiNPs) have been shown to decrease both the quantity and quality of sperm. Ren,et al. (36) reported that SiNPs led to the differential expression of miRNAs in GC-2spd cells. Exposure to low-dose SiNPs promotes autophagy through miRNA-494 targeting of AKT, thereby activating the AMPK/TSC/mTOR pathway. In addition, SiNPs suppress spermatogenesis by inhibiting miR-450b-3p, resulting in the upregulation of cytoskeletal proteins. Additionally, SiNPs induce apoptosis by activating the Hippo pathway and enhancing the traction force and adhesion between GC-2spd cells (33). Microcystin (MC)-LR is a cyclic heptapeptide that exhibits strong toxicity to the reproductive system. Specifically, it decreases sperm quality by affecting the spermatogonia. In a previous study, the expression of 25 miRNAs linked to spermatogenesis was significantly altered in GC-1 cells treated with MC-LR, with miR-96 being the most downregulated. miR-96 upregulation promotes cell viability by targeting DAZAP2 (37). Another study showed that miR-541expression is significantly higher in both GC-1 cells and mouse testes following exposure to MC-LR. Upregulation of miR-541 leads to a reduction in the expression of p15 and murine double minute 2 (MDM2), thereby promoting the activation of p53 and MC-LR-mediated cell apoptosis (15).

Spermatogenesis-related miRNAs are vulnerable to polycyclic aromatic hydrocarbons (PAHs). Urinary 2-OHFlu and 2-OHPh are associated with reduced levels of miR-34c in seminal plasma. In addition, urinary 9-OHPh is associated with reduced levels of miR-106a in seminal plasma, which, in turn, is positively associated with the concentration, count, total motility, and progressive motility of sperm (23). BPA can negatively affect sperm quality, concentration, and morphology. The expression of miR-let-7a/-7b/-7c was significantly higher, and that of miR-518f was significantly lower in the seminal plasma of men with azoospermia than in healthy men. Additionally, BPA levels were significantly and positively associated with miR-let-7a/-7c levels and negatively associated with miR-518f levels (29).

## 3 miRNAs and abnormal sperm quantity

Abnormal sperm quantity owing to primary testicular disturbances can manifest as azoospermia and OS in seminal phenotypes. Azoospermia is defined as the complete absence of sperm in the ejaculated semen and can be further classified into azoospermia (OA) and NOA. NOA are divided into three types based on the histological characteristics of the testis: Sertoli cell-only syndrome (SCOS), spermatogenic arrest at different stages of germ cell maturation (spermatogonia, spermatocytes, and spermatids), and hypospermatogenesis.

Several studies have evaluated the roles of miRNAs in patients withinfertility and azoospermia. Lian et al. compared the expression of miRNAs between testicular samples from men with NOA and those from fertile men using a microarray platform and identified 19 upregulated and 154 downregulated miRNAs (38). Similarly, Wu et al. (39) screened for differentially expressed miRNAs between 100 men with NOA and 100 fertile men using a TaqMan Low-Density Array. They determined that the expression of miR-141/-429/-7-1-3p was significantly higher in seminal plasma samples from men with NOA than in those from fertile men. Furthermore, the DNA hypomethylation-induced activation of miR-141 and miR-429 is a relatively common molecular occurrence in patients with NOA. This suggests that suppression of these two miRNAs may be linked to spermatogenesis. Zhang et al. (40) analyzed the miRNA expression profiles of testicular biopsies from men with NOA and healthy men using microarray technology and identified 83 upregulated and 46 downregulated miRNAs in samples collected from men with NOA. In addition, the upregulation of miR-370-3p/-539-5p/-10b-3p/-22-5p and the downregulation of miR-34b-5p/-31-5p/-516b-5p/-122-5p were verified via qRT-PCR. Fang (41) examined miRNA profiles of testicular tissues from patients with successful sperm retrieval (SSR and USR, respectively). Compared to SSR group, the USR group had 13 upregulated and 167 downregulated miRNAs. A total of 86 miRNAs were completely absent in the USR group but were highly expressed in the SSR group. The expression of hsa-34c-5p/-22-3p/-29b-3p was comparable between seminal plasma and testicular tissue samples, suggesting that these miRNAs are potential noninvasive biomarkers for predicting testicular sperm retrieval prior to micro-TESE. The downregulated miRNAs in USR were mapped to the clusters related to spermatogenesis (cluster hsa-miR-515, cluster hsa-miR-290, cluster hsa-miR-181, and cluster miR-34b/c), located on chromosomes 19, X, 17, 11, 7, and 5. Abu-Halima et al. (42) examined the miRNA expression profiles of patients with different histopathological patterns: SCOS, mixed atrophy (MA), germ cell arrest (GA), and normal spermatogenesis (N). A total of 46 miRNAs exhibited differential expression compared to the N group. In addition, the differential expression of hsa-mir-34b*/-34b/-34c-5p/-449a/-449b* was validated using qRT-PCR (43). MiRNAs are involved in apoptosis, proliferation, and differentiation. Zhuang et al. (44) reported that 93 miRNAs and 4172 mRNAs are differentially expressed in the testicular tissues of patients with NOA and OA. These differentially expressed genes were primarily associated with spermatogenesis, meiosis, the cell cycle, and the development of secondary male sexual characteristics. Sabetian (45) screened for the potential pathogenic miRNA–target gene pairs and lncRNA–miRNA pairs associated with NOA using a microarray dataset. A total of 74 mRNAs, 14 miRNAs, and 10 lncRNAs were found to be differentially expressed in the testicular tissues of men with NOA and fertile men. miR-509-5p and miR-27b-3p were found to interact with polo-like kinase 1 (PLK1) and CRISP2, respectively. Zhang et al. (46) investigated the miRNA expression profiles of seminal plasma collected from men with NOA with different histopathological patterns and seven fertile men using sequencing. Compared to fertile men with normozoospermia, men with SCOS had 78 upregulated and 132 downregulated miRNAs, whereas patients with spermatogenesis arrest (SA) had 32 upregulated and 90 downregulated miRNAs. The expression of hsa-miR-34c-5p was significantly lower in seminal plasma samples of men with NOA than in those of fertile men, suggesting that miR-34c-5p is a potential non-invasive biomarker for diagnosing NOA and distinguishing different pathological subtypes of NOA. The expression of miR-509-5p/-122-5p/-34b-3p/-34c-5p was lower in seminal plasma samples from patients with OA, NOA, and Klinefelter syndrome (KS) than in control individuals. In addition, a weak negative correlation was observed between follicle-stimulating hormone (FSH) levels and miR-509-5p expression in patients with NOA (47). Yao et al. (48) analyzed miRNA expression profiles using a microarray platform and determined that 174 miRNAs were differentially expressed in the Sertoli cells (SCs) of patients with SCOS and OA. The expression of miR-133b was higher in SCs derived from patients with SCOS than in SCs derived from patients with OA. miR-133b enhances SCs proliferation by targeting GLI3 and enhancing the expression of cyclin B1 and cyclin D1. Song et al. (49) characterized the protein and miRNA constituents of exosomes derived from human testicular endothelial cells (HTEC-Exos) and investigated their effects on spermatogenesis. Thirty miRNAs were shown to be associated with male reproductive disorders, such as azoospermia and abnormal spermatogenesis.

MiR-210 is highly expressed in the testes of men with NOA and participates in spermatogenesis by targeting IGF2 in male infertility (50). The expression of miR-34c-5p/-122/-146b-5p/-181a/-374b/-509-5p/-513a-5p is markedly low in azoospermia but high in asthenozoospermia (51). MiR-188-3p expression was significantly decreased in patients with OA and NOA compared to that in control individuals. miR-188-3p downregulation, induced by reduced histone acetylation, upregulates MLH1 expression and contributes to apoptosis in spermatogenic cells of patients with azoospermia (52). The expression of LC3 and beclin-1 was upregulated in the testes of patients with NOA and negatively associated with the expression of miR-188-3p. miR-188-3p may participate in autophagy by regulating its target gene, ATG7, thereby affecting the development of NOA (53). MiR-31-5p is highly expressed in the testis and epididymis, and its altered expression can be determined from both extracellular vehicles (EVs) and whole seminal plasma to distinguish obstructive from secretory azoospermic samples (54). The expression of hsa-miR-27a-3p was higher in men with NOA than in men with OA. Hsa-miR-27a-3p can mislead spermatogenesis and contribute to the development of male infertility by downregulating *KDM3A* directly and *TNP1* and *PRM1* indirectly (55). The expression of miR−192a in seminal plasma and testicular tissues was higher in men with varicoceles without spermatozoa in the ejaculate after surgery than in men with varicoceles with spermatozoa after surgery and in control men. Therefore, seminal plasma miR−192a may serve as a useful diagnostic marker for NOA, and men with varicoceles may benefit from varicocelectomy (56). The expression level of miR-202-5p/-34c-5p/-10b/-191/-126 were lower in patients with SCOS than in healthy individuals. MiR-202-5p is localized in the SCs of men with normal spermatogenesis but not in those of men with SCOS (57). miR-202-3p/-629-5p expression was significantly lower in men with azoospermia than in those with normozoospermia. In addition, the expression of miR-370-3p is significantly higher in the semen of men with azoospermia without sperm cells in the testes (58). Four miRNAs, hsa-miR-34b-3p, hsa-miR-34c-3p, hsa-miR-3065-3p, and hsa-miR-4446-3p, were identified as potential biomarkers for accurate assessment of spermatogenesis in the testes prior to performing micro-TESE in patients with NOA. Upregulated expression of hsa-miR-34b-3p and hsa-miR-34c-3p indicated normal spermatogenesis and increased the likelihood of sperm retrieval using micro-TESE. However, upregulated expression of hsa-miR-3065-3p and hsa-miR-4446-3p indicated impaired spermatogenesis and a decreased the likelihood of retrieving viable sperm (59). Hsa-miR-506, hsa-miR-507, and hsa-miR-510 are X-linked miRNAs. Rs1447393, located near hsa-miR-510, may function as a potential protective factor against NOA, whereas rs5951785, located near hsa-miRNA-506 and hsa-miR-507, may increase the risk of NOA by inhibiting cell proliferation and inducing cell apoptosis (60). The expression of miR-19b and miR-let-7a was significantly higher in men with NOA than in fertile men and was reproducible and stable in seminal plasma. Aberrant expression of miR-19b and miR-let-7a may indicate spermatogenic failure (61). The expression of miR-20a-5p is significantly higher in the blood plasma of men with NOA than in fertile men. In addition, it was negatively associated with serum total testosterone (TT) levels and right and left testicular size, and positively associated with FSH and luteinizing hormone (LH) levels (62). The expression of miR-202-3p is higher in SCs derived from patients with SCOS than in those derived from OA patients with normal spermatogenesis. miR-202-3p participates in spermatogenesis by regulating the proliferation, apoptosis and synthesis of SCs by targeting LRP6 and cyclin D1 in the Wnt/β-catenin signaling pathway (63).

Oligospermia is defined as a sperm cell count <15 million/mL in a male ejaculate. Several miRNAs are involved in Oligospermia development. MiR-371a-3p has been detected in the testis parenchyma and semen, and its levels have been associated with sperm concentration (64). The expression of miR-371a-3p is significantly lower in the unprocessed ejaculates of patients with Oligospermia than in those of healthy individuals. It is significantly associated with total sperm count but not with sperm motility, sperm morphology, or days of abstinence (65). MiR-122, miR-181a and miR-34c-5 are downregulated in the seminal plasma of men with varicoceles and oligoasthenoteratozoospermia. The expression of these three miRNAs is associated with increased varicocele grade and bilaterality and is positively associated with sperm concentration, total sperm motility and normal sperm morphology (66). Expression levels of miR-let-7a and miR-7-1-3p/-141/-200a/-429 were significantly higher in the sperm and seminal plasma of infertile men than in those with normozoospermia. In addition, it was significantly negatively associated with sperm concentration. The expression of miR-15b/-34b/-122 is significantly downregulated in men with infertility and is positively associated with sperm concentration (5). The expression of miR-122-5p is lower in the sperm and exfoliated cells of patients with oligospermia than in those of healthy individuals and is positively associated with sperm density (67). The expression patterns of miR-10b/-135b/-10a/-135a were normal in normal sperm. The expression of miR-135a is high in patients with asthenospermia, oligospermia, and asthenospermia-oligospermia, and miR-135b has been identified as a robust biomarker for distinguishing between patients with AS-OS, healthy individuals, and patients with other types of infertility (68). In a previous study, miR-106b∼25KO testes exhibited reduced size, oligospermia, and altered spermatogenesis. Severely disrupted histological characteristics of the testes and significantly reduced fertility were observed in miR-17∼92+/-; miR-106b∼25-/- double mutant mice, suggesting that miR-17-92 and mir-106b-25 are essential for maintaining testis homeostasis and male fertility (69). rs4938723, located within miR-34b/c, showed significantly different genotypic and allelic distributions between patients with oligospermia and control. In particular, the CC genotype of rs4938723 is predominant in patients with oligospermia compared to controls (70). The expression of miR-21/-22 is significantly higher and that of ER-β is lower in patients with oligospermia than in healthy individuals. These genes indirectly participate in spermatogenesis by regulating estrogen receptors, and may serve as potential diagnostic and prognostic biomarkers for male infertility (71). The expression of miR-23a/b-3p was high and that of ODF2 and UBQLN3 was low in patients with oligoasthenozoospermia. These genes are negatively associated with sperm count, motility and morphology (72). MiR-34 family members are markers of low semen concentrations and play a crucial role in spermatogenesis. The expression of miR-34b-5p is lower in men with AZ and oligospermia than in those with normozoospermia (73). Salas-Huetos et al. (13) analyzed the miRNA expression profiles of spermatozoa from three groups of infertile men and one group of fertile men. Eighteen miRNAs were differentially expressed in the oligospermia group; in particular, hsa-miR-335-5p, hsa-miR-885-5p, and hsa-miR-152-3p were associated with sperm concentration. The expression of miR-765 and miR-1275 is significantly higher, and that of miR-15a is lower in extracellular macrovesicles from subfertile patients with oligoasthenozoospermia than in those from controls (14). In one study, miR-34b/-34b*/-15b/-34c-5p/-122/-449a/-1973/-16/-19a exhibited higher fold changes in patients with oligoasthenozoospermia than in control individuals with normozoospermia, and were associated with sperm count (9). The miRNAs associated with azoospermia and oligospermia in patients with infertility are summarized in **Table 2**.

**Table 2:** miRNAs related to abnormal sperm quantity.

Ref	miRNA	Studied patients	Position	miRNA measurement	Comparison	Variation	Target genes	Effects on spermatogenesis
(58)	miR-202-3p;	AZ=28; N=18	T/Sp	Sequencing; qRT-PCR;	AZ vs N	down	NA	NA
(54)	miR-31-5p	OA=43; NOA=43; OS=5; N=13	Sp	qRT-PCR	NOA vs OA	up	NA	NA
(45)	miR-509-5p; miR-27b-3p	NOA=27; N=4	T	Tissue Microarray	NOA vs N	up	*Plk1;* *Crisp2*	NA
(53)	miR-188-3p	NOA=16; N=16	T	qRT-PCR;	NOA vs N	down	*Atg7*	Involved in NOA by regulating autophagy.
(59)	miR-34b-3p; miR-34c-3p;	NOA=22; N=6	Sp	qRT-PCR	NOA vs N	up	NA	NA
	miR-3065-3p; miR-4446-3p					down		
(68)	miR-10a; miR-10b; miR-135a; miR-135b	AZ=30; OS=30; OAZ=30; N=30	S/Sp	qRT-PCR	AZ/OS/OAZ vs N	up	NA	NA
(46)	miR-34c-5p; miR-202-3p;	NOA=13; N=7	Sp	RNA-Seq; qRT-PCR	NOA vs N	down	NA	NA
	miR-141-3p					up		
(72)	miR-23a/b-3p	OAZ=46; N=46	S	qRT-PCR;	OAZ vs N	up	*Odf2;* *Ubqln3*	Negative correlate with sperm count.
(73)	miR-34b-5p	AZS=50; OS=22; N=43	Sp	qRT-PCR	AZS/OS vs N	up	NA	Positive correlate with semen concentration.
(40)	miR-10b-3p; miR-370-3p; miR-539-5p; miR-22-5p	NOA=39; N=38	T	Microarray; qRT-PCR	NOA vs N	up	NA	NA
	miR-34b-5p; miR-516b-5p; miR-122-5p;					down		
(47)	miR-509-5p; miR-122-5p; miR-34b-3p; miR-34c-5p	NOA=60; KS=40 OA=60; N=40;	Sp	qRT-PCR	NOA/KS /OA vs N	down	NA	NA
(55)	miR-27a-3p	NOA=19 OA=11	T	qRT-PCR	NOA vs OA	up	KDM3A	NA
(62)	miR-20a-5p	NOA=14; N=10	B	qRT-PCR	NOA vs N	up	NA	NA
(5)	let-7a; miR-7-1-3p; miR-141; miR-200a; miR-429	AZS=10; OS=10; N=10	S/Sp	qRT-PCR	AZS/OS vs N	up	NA	negative correlate with sperm concentration
	miR-15b; miR-34b; miR-122					down		positive correlate with sperm concentration
(70)	miR-34b/c; CC of rs4938723	AZ=144; OS=273; N=234	B	qRT-PCR	AZ/OS vs N	up	NA	NA
(63)	miR-202-3p	SCOS=20; OA=20;	T	qRT-PCR	SCOS vs OA	up	*Lrp6;* *cyclin D1*	NA
(64)	miR-371a-3p	OS=3; N=11	S	qRT-PCR	OS vs N	down	NA	NA
(65)	miR-371a-3p	OS=12; N=26	Sp	qRT-PCR	OS vs N	down	NA	positive correlate with sperm count
(41)	miR-34c-5p; miR-22-3p; miR-29b-3p	SSR=5; USR=5	T	RNA-Seq; qRT-PCR;	USR vs SSR	down	*Prnd*	NA
(56)	miR-192a	NOA-S=27; NOA-US=33; N=30	Sp/T	qRT-PCR;	NOA-US vs NOA-S/N;	up	NA	Influences proliferation and apoptosis of cells.
(74)	miR-34b	NA	T	qRT-PCR; Microarray	MB vs IB	up	*Map2k1*	Influences proliferation, cell cycle and proptosis of SCs.
(52)	miR-188-3p	NOA=8; OA=8; N=8	T	qRT-PCR;	NOA/OAvs N	down	*Mlh1*	Influences spermatogenic cells apoptosis
(60)	miRNA-506/507;	NOA=1109; N=1193	NA	miRNAs Sequencing; qRT-PCR	NOA vs N	up	*Gli3* *Pik3c2A* *Adam17* *Prdx1*	Influences cells proliferation and apoptosis.
	miRNA-510					down		
(48)	miR-133b; miR-204-5p; miR30e-5p; miR-4270; miR-129-2-3p; miR-101-3p miR-202-3p; miR-195-5p; miR-664b-3p; miR-497-5p; miR-34b-5p; miR-513a-5p;	NA	T	qRT-PCR; Microarray	SCOS vs OA	up	miR-133b- *Gli3*	Influences SCs proliferation
	miR-221-3p; miR-409-5p; miR-1290; miR-155-5p; miR-31-3p; miR-7-5p; miR-362-5p; miR-199b-5p miR-493-5p; miR-296-5p;					down		
(66)	miR-122; miR-181a; miR-34c5	VxF=43; N=52 OAT=62; OAT+Vx=63;	Sp	qRT-PCR	OAT+Vx vs VxF/OAT/N	down	NA	Influences proliferation and oxidative stress.
(44)	miR-563; miR-375; miR-521; miR-125a-3p; miR-34c-3p	NOA=4; OA=3	T	Microarray; qRT-PCR	NOA vs OA	down	NA	NA
(57)	miR-202-5p; miR-10b; miR-191; miR-34c-5p; miR-126-5p	SCOS=5; N=13	T	qRT-PCR; miRNA ISH	SCOS vs N	down	NA	NA
(13)	miR-335-5p; miR-885-5p; miR-152-3p	AZS=10; TZ=10; OS=10; N=10	S	qRT-PCR	OS vs N	up	NA	Negative correlate with sperm concentration.
(42)	miR-34c-5p; miR-449a; miR-34b*/34b; miR-449b; miR-517b; miR-129-3p	NOA=40; N=16	T	qRT-PCR; Microarray	NOA vs N	down	NA	NA
(71)	miR-21; miR-22	OS=43; N= 43	S	qRT-PCR	OS vs N	up	ERβ	Negative correlate with sperm count.
(43)	miR-429	NOA=40; OA=16; N=90	T/S	qRT-PCR; Microarray	NOA/OA vs N	up	NA	NA
	miR-34b; miR-34c-5p; miR-122					down		
(39)	miR-141; miR-429; miR-7-1-3p	NOA=100; N=100	Sp	qRT-PCR; TLDA chips	NOA vs N	up	NA	NA
(9)	miR-122; miR-34c-5p; miR-34b; miR-16;	AZS=9; OAZ=9; N=9	Sp	Microarray; qRT-PCR	A ZSvs N OAZ vs N	down	NA	NA
	miR-141; miR-200a					up		
(61)	miR-19b; let-7a	NOA=96; OS=96; N=96	T/Sp	qRT-PCR	NOA vs N OS vs N	up	NA	Influences proliferation of germ cells.
(51)	miR-34c-5p; miR-122; miR-374b; miR-181a; miR-513a-5p; miR-509-5p	NOA=118; AZS=137; O=34; N=168	Sp	Solexa sequencing; qRT-PCR	NOA vs N	down	NA	NA
					AZS vs N	up		
(38)	miR-302a; miR-491-3p	NOA=3; N=2	T	Microarray; qRT-PCR;	NOA vs N	up	NA	NA
	miR-383; miR-520d-3p					down		

AZS, Asthenozoospermia; OAZ, Oligoasthenozoospermia; OAT, Oligoasthenoteratozoospermia; OS, Oligozoospermia; N, normal spermatogenesis; S, Sperm; SP, Seminal plasm; T, Testis; TZ, Teratozoospermia; NOA-S, NOA with clinical varicocele patients following microsurgical subinguinal varicocelectomy with successful ejaculatory spermatozoa; NOA-US, NOA with clinical varicocele patients following microsurgical subinguinal varicocelectomy with unsuccessful ejaculatory spermatozoa; MB, mature bull; IB, immature bull; SSR, NOA patients with successful sperm retrieval; USR, NOA patients with unsuccessful sperm retrieval; B, Blood; Vx, varicocele; VxF, Fertile normozoospermic men with Vx; OAT+Vx, infertile OAT men with Vx; Ref, Reference.

## 4 Mechanism of miRNA involved in spermatogenesis

Spermatogenesis is a multi-step process of division and differentiation of germ cells regulated by FSH and LH. It takes place in the seminiferous tubules of the testes and consists of three stages: proliferation and differentiation of spermatogonia, maturation division of spermatocytes, and spermiogenesis (sperm formation). Spermatogenesis is a highly coordinated process that relies on the regulation of various somatic cells, including SCs, mesenchymal cells, and peritubular myoid cells. SCs play a crucial role in this process by closely interacting with developing germ cells. They provide essential support and nourishment to ensure the proper progression of spermatogenesis. Many studies have shown that miRNAs are involved in various stages of spermatogenesis (**Graphical Abstract**).

### 4.1 miRNAs and spermatogonia stem cells (SSCs)

Spermatogenesis is a highly organized process that requires a balance between the proliferation and differentiation of SSCs. SSCs reside in a unique microenvironment or ‘niche’ in the testes and are the foundation of spermatogenesis. Intrinsic factors and extrinsic signals play a crucial role in maintaining a delicate balance between SSC proliferation and differentiation, which ensures a continuous supply of differentiating spermatogonia while preserving an adequate population of stem cells (75) and ensuring the lifelong production of spermatozoa. miRNAs are important endogenous regulators of this process in spermatogenesis (76).

Under steady-state conditions, spermatogenesis is initiated when the undifferentiated progenitor spermatogonia transition to a differentiating state, eventually resulting in sperm formation. KIT protein was detected on the surface of differentiating SSCs but not on the surface of undifferentiated SSCs. The KIT receptor is a hallmark of the SSC transition from an undifferentiated to a differentiating state. miR-221 and miR-222 repress the transition of SSCs from a KIT^–^ to a KIT^+^ state, play an important role in regulating the undifferentiated state of SSCs, and influence the maintenance of stem cell properties (77). miR-202 plays an important role in maintaining stem cell properties of cultured mouse SSCs. In previous study, KO of miR-202 in mice facilitated the initiation of SSC differentiation and meiosis and led to aberrant and premature expression of SYCP3, STRA8, and DMRT6 (78). DMRT6 was a direct target of miR-202-5p (79). The inhibition of SSC differentiation by downregulating Plzf expression and upregulating Stra8, Dazl, and Sycp3 expression (80). Inhibition of miR-30a-5p triggers the differentiation of neonatal mouse SSCs (76), whereas its overexpression increases cell viability and promotes antiapoptotic and antioxidant activities during the freeze-thaw process of SSCs (81). SC-derived exosomal miR-30a-5p promotes the proliferation and differentiation of SSCs by regulating the MAPK signaling pathway through Zeb2/Fgf9 (82).

In a previous study, the proliferation or undifferentiation marker ID4 was considerably upregulated in SSCs treated with an miR-30 mimic, suggesting that miR-30 regulates the self-renewal capability of SSCs (83). miR-31-5p inhibits proliferation and DNA synthesis and increases early and late apoptosis of human SSCs by targeting JAZF1 and cyclin A2 (84). miR-31 directly targets Stra8 to inhibit meiosis and spermatogenesis in chicken SSCs (85). miR-106b-5p improves the proliferative capacity of SSCs. Upon stimulation with miR-106b-5p, SSCs exhibit high expression levels of TGF-β1, which is a well-known regulator of epigenetic modifiers and downstream genes (86). miR-20 and miR-106a are preferentially expressed in mouse SSCs and are largely confined to the spermatogonia along the basement membrane of adult mouse seminiferous tubules. Overexpression of miR-20 and miR-106a promotes the proliferation, renewal, and DNA synthesis of SSCs, leading to the accumulation of undifferentiated SSCs *in vivo* and a decrease in the number of sperm (87). In addition, miR−106b enhances the differentiation of human mesenchymal stem cells (MSCs) into SSCs (88) and miR-106b-5p regulates the reprogramming of SSCs into iPSC-like cells (89). The expression of miR-122-5p is higher in spermatogonia derived from patients with OA than in those derived from patients with NOA. miR-122-5p stimulates cell proliferation and DNA synthesis while suppressing early apoptosis in SSCs by targeting CBL and competing with the lncRNA CASC7 (90). miR-34c is specifically expressed in the testes and has the highest expression levels in spermatogenic cells of sexually mature mice. Overexpression miR-34a promotes meiosis in SSCs both *in vitro* and *in vivo* (91). miR-34c inhibits the expression of Nanos2 in GC-1 cells. Dysregulated miR-34c/Nanos2 expression disrupts the balance between the self-renewal and differentiation of SSCs, eventually damaging spermatogenesis in cryptorchid testes (92). miR-22-5p modulates SSCs self-renewal by targeting EZH2. Elevated levels of miR-22-5p hinder SSCs proliferation, increase the apoptotic rate, and decrease the expression of SSC marker proteins (93). miR-10b is highly expressed in mouse SSCs and plays a role in regulating SSC self-renewal through Kruppel-like factor 4 (94). The expression of miR-10a is high in different types of germ cells and low in SCs, with the highest expression detected in postnatal 7 (P7) testes. Overexpression of miR-10a in male germ cells hinders the initiation of meiosis, resulting in severe testicular atrophy and male sterility in adulthood (95). miR-100 is predominantly expressed in undifferentiated murine spermatogonia, including SSCs, and promotes the proliferation of SSCs *in vitro* by regulating Stat3 indirectly (96). miR-322 is highly expressed in SSCs and regulates the self-renewal and differentiation of SSCs through the WNT/β-catenin signaling by targeting Rassf8 (97). miR-204 plays a role in regulating the proliferation and self-renewal of dairy goat SSCs by interacting with Sirt1 (98). miR-135a contributes to SSC maintenance by modulating FoxO1 activity. Low expression of miR-135a in undescended testes is associated with cryptorchidism (99). Furthermore, miR-224 exhibits significant expression levels in mouse SSCs and plays a crucial role in their self-renewal and differentiation by targeting DMRT1 through WNT/b-catenin signalling (100). SC-derived exosomal miR-486- 5p acts as a communication molecule between SCs and SSCs and modulates SSCs differentiation through PTEN (101). Low miR-181a levels are associated with defects in spermatogenesis in testicular samples obtained from patients with azoospermia. Knockdown of miR-181a attenuated spermatogonia proliferation and G1/S-phase arrest and increased S6K1 (102). CEP55 is primarily expressed in the testes, and its levels are significantly higher during normal spermatogenesis than during maturation arrest. miR-449a suppresses mouse spermatogonia proliferation by inhibiting CEP55 (103).

### 4.2 miRNAs and spermatocytes in meiosis

Initiation of the meiotic phase of spermatogenesis requires male germ cells to exit mitosis and promptly express meiosis genes. Meiosis disorder and recombination defects can lead to male infertility. Several studies have demonstrated that specific miRNAs regulate meiosis. In one study, the miR-449 cluster and miR-34b/c, which have similar expression patterns, were predominantly expressed in mouse testes. Their expression levels were significantly upregulated during the onset of meiosis, testicular development, and adult spermatogenesis, with the highest levels detected in meiotic (spermatocytes) and postmeiotic (spermatids) male germ cells. Simultaneous KO of the miR-449 and miR-34b/c clusters leads to sexual dimorphism and infertility (104). miR-34b/c and miR-449 clusters regulate spermatogenesis together by targeting the E2F-pRb pathway. KO of either the miR-34 or miR-449 cluster leads to no apparent defects in male germ cell development. miR-34b/c and miR-449 double-KO males mice exhibit chromatin condensation and flagellar defects and are infertile owing to severe spermatogenic disruptions and OAT (105). miR-34c is detectable in mouse pachytene spermatocytes and is highly expressed in spermatids. Inhibition of miR-34c leads to a reduced spontaneous apoptotic rate and an elevated Bcl-2/Bax ratio, suggesting that miR-34c enhances apoptosis in murine male germ cell by targeting ATF1 (91). Germ cell-specific miRNAs (miR-741-3p, miR-871-3p, and miR-880-3p) are X-chromosome-linked miRNAs (XmiRs). Spermatogenesis is halted at the meiosis stage in the abnormal seminiferous tubules of ΔXmiR mice (106). miR-18, a member of the miR-17-92 cluster, is highly expressed in spermatocytes. It targets heat shock factor 2 (Hsf2), an essential transcription factor for spermatogenesis and is required for chromatin organization and sperm head structure (107). miR-188-3p is prominently expressed in human testis-specific cells, such as spermatogonia and spermatocytes, and is detectable during the meiotic phase of mouse cells. miR-10a showed the highest expression in P7 testes, which gradually declined to adult testes (P60), suggesting that miR-10a may perform preferential functions in germ cell development. Overexpression of miR-10a in germ cells results in meiotic arrest and infertility due to its inability to repair double-strand breaks and abnormal spermatid differentiation (95). miR-181a is highly expressed during mouse spermatogenesis and its KO attenuates cell proliferation and G1/S-phase arrest by increasing S6K1. Decreased miR-181a expression is associated with spermatogenesis defects in the testes of patients with azoospermia (102). miR-202, a member of the let-7 family, plays a crucial role in spermatogenesis, and its KO in mice inhibits spermatocyte apoptosis and disrupts the zygonema-to-pachynema transition. Additionally, various processes during meiotic prophase I, such as synapsis and crossover formation, are disturbed, and intersister chromatid synapses have been observed (79). Sethi et al. (108) identified differentially expressed miRNAs among pachytene spermatocytes, round spermatids, and mature rat sperm using high-throughput transcriptomic sequencing. A total of 149 miRNAs were restricted to primary spermatocytes as they were not detected in the post-meiotic cells. The 10 most abundant miRNAs specific to this stage were miR-12204-5p, miR-511-3p, miR-495-3p, miR-7–1-3p, miR-7232-3p, miR-301a-3p, miR-449c-3p, miR-93-3p, miR-503-5p, and miR-6329. These spermatocyte-specific miRNA target genes are involved in meiosis regulation. Liu et al. (109) effectively isolated human spermatogonia, pachytene spermatocytes, and round spermatids using STA-PUT velocity sedimentation. The miRNA profiles of these cells were investigated using a microarray platform. In total, 32 miRNAs were significantly upregulated and 78 miRNAs were downregulated in human spermatogonia and pachytene spermatocytes, suggesting that these miRNAs are involved in meiosis and mitosis. The differentially expressed miRNAs included let-7a-5p, miR-125b-5p, miR-126-3p, miR-100-5p, miR-34c-5p, and miR-34b-5p. DNA damage in male germ cells impairs spermatogenesis and reduces fecundity. DNA damage is aberrantly increased in pachytene spermatocytes in the testicular tissues of maturation arrest (MA) males. miR-383 is predominantly expressed in spermatogonia and primary spermatocytes, where meiosis occurs, and is downregulated in the testes of men with MA. miR-383 disrupts the phosphorylation of H2AX by targeting PNUTS and independently induces cell cycle arrest (110). In one study, either *Dgcr8* or *Dicer* was conditionally knocked out in mice (conditional KO [cKO] mice) to assess the effects of their miRNAs on spermatocytes during meiosis. No mature sperm were observed in *Dicer*-cKO and *Dgcr8*-cKO mice. Frequent chromosomal fusions observed in prophase I spermatocytes in both *Dgcr8*- and *Dicer*-cKO germlines indicated that one or more miRNAs regulate crucial aspects of chromosomal integrity during meiosis. The expression levels of miR-1, miR-183, and miR-16 are lower in *Dgcr8-* and *Dicer*-cKO-spermatocytes than in WT spermatocytes (111).

SiNPs induce reproductive toxicity by inducing cell cycle arrest, inhibiting cell proliferation, and promoting the apoptosis of spermatocytes. In one study, the expression of 15 miRNAs was altered in spermatogenic cells exposed to SiNPs for 30 passages. Among these 15 miRNAs, miR-138 was upregulated and whereas miR-2861 was downregulated. miRNA-2861 functions through the RIPK1 signalling pathway and may serve as a biomarker for SiNP-induced spermatocyte apoptosis (20). SiNPs can inhibit miR-450b-3p expression and activate mitochondrial apoptosis signalling pathways by regulating MTCH2 in spermatocytes, thus inducing reproductive toxicity (112). The cytotoxicity of SiNPs -stimulated GC-2spd cells depend on the dysregulation of multiple miRNAs that regulate DNA replication and fatty acid metabolism (113).

### 4.3 miRNAs and spermatids

During the early stages of spermiogenesis, haploid spermatids undergo significant structural reorganization, resulting in significant morphological transformations. This phase of spermatogenesis encompasses the condensation and remodelling of nuclear chromatin and shape, development of acrosomes, elimination of residual cytoplasm, and assembly of the sperm flagellum. Ultimately, the fully developed and elongated spermatids are released as spermatozoa through a process known as spermiation (114). Transition nuclear proteins (TNPs) are highly basic proteins rich in arginine and lysine. They possess strong affinity for DNA and are specifically expressed in post-meiotic haploid spermatids. These proteins are displaced from condensing chromatin and replaced by protamine (PRMs), which serves as a primary nuclear proteins in condensed spermatids and mature sperm. Precise timing and stage-specific translation of TNPs are crucial for the proper development of round spermatids into fully mature spermatozoa and for the maintenance of male fertility. TNPs and PRMs are crucial for the process of sperm maturation. The presence and regulation of transition proteins (TP) and PRM are essential for the elongation of spermatids and development of mature sperm. Testis-specific miR-469 plays a pivotal role in the regulation of sperm cell development and maturation by repressing the translation of TNP2 and PRM2 mRNA (115). miR-122 is upregulated in the abnormal sperm of men with infertility and is closely associated with the occurrence of abnormal spermatozoa. miR-122 acts as a suppressor of TNP2 expression, thereby influencing the transformation of induced pluripotent stem (iPS) cells into spermatozoa-like cells (116). In one study, 173 miRNAs exhibiting differential expression in human pachytene spermatocytes and round spermatids were identified. These findings suggested that these miRNAs play crucial roles in male meiosis and spermiogenesis. Among them, miR-206 is upregulated in round spermatids compared to in pachytene spermatocytes, suggesting that it plays a regulatory role in spermiogenesis (109). miR-449 is predominant expressed in mouse testes and is primarily localized in spermatocytes and spermatids (104). Additionally, miR-34c is highly expressed in mouse pachytene spermatocytes and round spermatids in mouse testes, and appears to play an important role in the later stages of spermatogenesis by targeting activating transcription factor 1 (ATF1) (10). Deficiency of miR-34bc/449 not only impairs meiosis and hinders the final stages of spermatozoa maturation (11). miR-34bc^-^/^-^;449^-^/^-^ mice exhibit a decrease in the proportion of spermatozoa, elongating spermatids, and abnormalities in sperm morphology with the separation of spermatozoa heads and tails (11). In one study, miR-32, miR-184, miR-335, miR-140, miR-141, miR-1981, miR-202, miR-880, and miR-669c were downregulated in both KO and KI mice, whereas miR-150, miR-196a-2, miR-652, miR-146, miR-10b, miR-379, miR-122a, miR-26a, miR-27a, miR-127, and miR-328 were upregulated in both KO and KI mice and negatively regulated spermatid differentiation (117). Liu et al. (109) isolated human spermatogonia, pachytene spermatocytes and round spermatids and used a microarray platform to analyze the miRNA expression profiles of these cells. A total of 144 miRNAs were upregulated and 29 miRNAs were downregulated between pachytene spermatocytes and round spermatids, suggesting their potential involvement in mediating spermiogenesis. RT-PCR validated that the expression of miR-100-5p, miR-34c-5p and miR-34b-5p was higher and that of miR-206 was lower in human pachytene spermatocytes than in round spermatids. Elevated expression of miR-199-5p inhibited the formation of sperm flagella during spermiogenesis by negatively regulating the expression of Tekt1, eventually leading to sperm abnormalities in male allotriploid crucian carp (118).

### 4.4 miRNAs and SCs

SCs play a key role in supporting testicular structure. They create a specialized environment that supports and dictates spermatogenesis. Transition of SCs from an immature proliferative state to a mature non-proliferative state during puberty. Mature SCs are involved in the formation of the blood-testis barrier (BTB), protecting and providing nutrition for developing sperms, promoting the development of germ cells into spermatozoa, secreting multiple growth factors, providing morphogenetic support and a stable microenvironment for the development and maturation of spermatozoa, preventing toxins from entering the epithelium, and delivering energy to sperm. The quantity of SCs plays a crucial role in maintaining normal spermatogenesis. Abnormalities in the function or number of SCs can lead to impaired spermatogenesis, potentially resulting in male infertility. The proliferation of SCs is tightly regulated by various factors, including transcription factors, hormones, and noncoding RNAs. Certain miRNAs exhibit specific expression patterns in SCs and stromal cells, and play significant roles in testicular development, functional regulation, and spermatogenesis. Various miRNAs have been shown to regulate proliferation and apoptosis of SCs. Yao et al. (48) used a microarray platform to analyze the miRNA expression profiles of patients with SCOS and OA with normal spermatogenesis. The expression of 174 miRNAs was altered in human SCs. The expression of some miRNAs (hsa-miR-133b/-204-5p/-30e-5p/-4270/-129-2-3p/-202-3p/-195-5p/-664b-3p/-497-5p/-34b-5p/-513a-5p/-101-3p/-221-3p/-409-5p/-1290/-155-5p/-31-3p/-7-5p/-362-5p/-493-5p/-296-5p/-199b-5p) was verified using qRT-PCR, suggesting that these miRNAs are associated with the pathogenesis of SCOS. In particular, the expression of miR-133b was higher in SCs derived from patients with SCOS than in those derived from patients with OA. miR-133b enhanced the proliferation of human SCs by targeting GLI3. In addition, the expression of miR-4270 was significantly higher in SCs derived from patients with SCOS than in those derived from healthy individuals. miR-4270 regulated the proliferation and apoptosis of SCs in patients with SCOS by inactivating the NOTCH signalling pathway through the GADD45A gene. Silencing miR-4270 significantly enhanced the proliferation of human SCs and TM4 cells and upregulated the expression of CCNE1, CCND1, and CDK4 (119). miR-202-3p regulates the proliferation, apoptosis and synthesis function of human SCs by targeting LRP6 and cyclin D1 in the Wnt/b-catenin signalling pathway (63).

Immature SCs have proliferative capacity, and the number of mature SCs depends on the number of immature SCs. Therefore, the fate of immature SCs is crucial for spermatogenesis. miR-638 inhibits the growth of immature SCs by indirectly inactivating the PI3K/AKT pathway through the SPAG1 gene (120). miR-126 can stimulate cell proliferation and suppress apoptosis of immature porcine SCs by targeting the PIK3R2 gene through the PI3K/AKT signalling pathway, suggesting that miR-126/PIK3R2 and the PI3K/AKT signalling pathway play important roles in regulating porcine spermatogenesis by degerming the fate of immature SCs (121). miR-222 can suppress the growth of immature porcine SCs by targeting the GRB10 gene through inactivation of the PI3K/AKT signalling pathway (122). Inhibition of miR-362 promotes the entry of cells into the S phase and increases the expression of cell cycle-related genes such as c-MYC, CNNE1, CCND1, and CDK4, by targeting the RMI1 gene (123). miR-499 is primarily located in the basement membrane of the seminiferous tubules of prepubertal porcine testicular tissues. It promotes cell proliferation and inhibits apoptosis in immature porcine SCs by targeting the PTEN gene through the PI3K/AKT pathway (124). miR-130a has been demonstrated to regulate the expression of 91 proteins and multiple pathways, enhance the phosphorylation SMAD5 and contribute to SC proliferation and testis development *in vivo*. In addition, it promotes the growth of immature porcine SCs by activating SMAD5 through the TGF-β-PI3K/AKT signalling pathway (125). A previous study reported that miR-26a is differentially expressed in developing pig testicular tissues. miR-26a expression was significantly higher at 60 d after coitus, at 1 d, 30 d of age than at other stages. miR-26a inhibits proliferation and promotes apoptosis in porcine SCs by targeting the PAK2 gene (126) and suppresses autophagy in swine SCs by targeting ULK2 (127). miR-196a expression was significantly higher in immature porcine testes than in mature porcine testes. In addition, miR-26a suppresses autophagy in swine SCs by targeting ULK2 (127). In contrast, miR-196a expression was significantly higher in immature porcine testes than in mature porcine testes. miR-196a regulates the proliferation and apoptosis of immature SCs and inhibits the expression of RCC2 and ABCB9 (128). miR-125a-5p increases the proportion of TM4 SCs in the G1 phase and decreased the proportion of cells in the G2 phase. Additionally, it suppresses cell proliferation and promoted apoptosis in TM4 SCs via RAB3D (129).

Porcine SCs undergo two proliferation phases. The first phase occurs from birth to 1 month of age, whereas the second phase starts at the onset of puberty. During puberty, the role of SCs transitions from regulating testis formation to maintaining spermatogenesis, a process referred to as maturation or differentiation of SCs. Throughout SC maturation, ssc-miR-149 was consistently expressed, with levels in SCs being 15-fold higher than those in other cell types. Gene Ontology (GO) analysis revealed that ssc-miR-149 is involved in various biological processes, including innate immune responses, apoptosis, and signaling pathways. It is also enriched in cellular components such as the transcription factor complex and endomembrane system, and exhibits molecular functions such as signal transducer activity, GTPase activator activity, and FMN binding. Notably, ssc-miR-149 plays a critical role in SCs by targeting TRAF3 (130). Overexpression of miR-16 in swine SCs resulted in decreased WT p53-induced phosphatase 1 (WIP1) expression and increased expression of both P53 and phosphorylated P53. miR-16 regulates the proliferation of swine SCs by influencing the expression of these factors (131). miR-382-3p is transcribed at nearly 10 times higher levels in immaturely proliferating SCs. However, it is naturally downregulated in mature SCs when puberty commences. Decreased in miR-382-3p levels in testicular SCs during puberty are essential for functional maturity of SCs. This downregulation enables SCs to support germ cell division and promote robust sperm production during this stage of development. Interestingly, overexpression of miR-382-3 in mature SCs led to severe testicular impairment in mice. This impairment is characterized by non-obstructive oligospermia and complete infertility. These changes may be attributed to the decreased expression of important genes such as AR, WT1, GJA1, INHBB1, ESR1, CREB1, and PTEN (132). SCs are the exclusive targets of FSH in the testes. SCs undergo functional maturation after birth and play crucial roles in enhancing germ cell division and differentiation during puberty. An important aspect of SC maturation is the decreased expression of miR-92a-3p, which is induced by FSH. This decrease in miR-92a-3p expression is a prerequisite for the onset of spermatogenesis during puberty. Elevated expression of miR-92a-3p in post-pubertal testes results in the development of functionally compromised SCs, which impair BTB formation and induce the apoptosis of pre-meiotic germ cells, eventually leading to infertility (133). In one study, the expression of miR-181c/d was higher in the testicular tissues of 60-d-old boars than in those of 180-d-old boars. Overexpression of miR-181c/d in SCs and murine testes disrupted BTB function by altering the distribution of BTB-associated proteins at the SC-cell interface and F-actin organization. miR-181c/d represses SC proliferation and promotes apoptosis. In addition, it regulates SC survival and barrier function by targeting platelet-activating factor acetylhydrolase 1b regulatory subunit 1 (Pafah1b1) gene (134). miR-34c is one of the differentially expressed in immature and mature bovine testes. The expression of miR-34c was higher in adult bovine testes than in the immature testes of newborns. Overexpression of miR-34c increases the apoptotic rate of bovine testicular SCs while decreasing the proliferation of SCs and the levels of PCNA and secreted factors (GDNF, BMP4. and CXCL12) (135). miR-34b regulates the proliferation and apoptosis of primary SCs through the MEK/ERK signalling pathway. Transfection of primary SCs with miR-34b mimics inhibited cell proliferation, induced cell cycle arrest at the G2 phase, and decreased the expression of cell cycle-related genes, such as CCNB1, CDK1, CDC25C, and C-MYC. Moreover, overexpression of miR-34b enhances apoptosis, as evidenced by an increase in the expression of the pro-apoptotic genes P53 and BAX and a decrease in the expression of the antiapoptotic gene BCL2 (74).

### 4.5 Synergistic effect of different miRNAs in spermatogenesis

**Figure 1:**
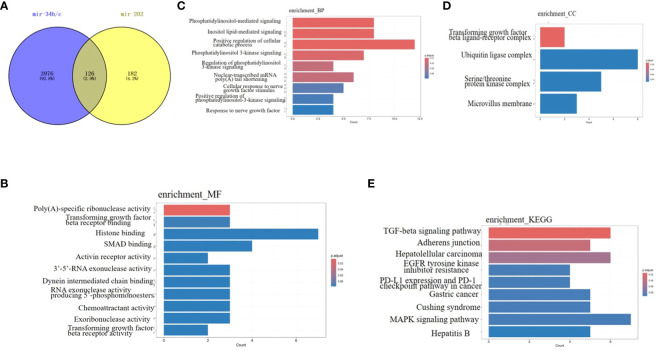
Bioinformatics analysis of miRNA-34b/c and miRNA-202 **(A)** The 126 common target genes of miRNA-34b/c and miRNA-202(Venn diagram). **(B)** Molecular functions. **(C)** Biological process. **(D)** Cell component. **(E)** Signalling pathways.

Each miRNA has several target mRNAs with multiple binding sites for the same or different miRNAs. Therefore, different miRNAs may exhibit synergistic effects during the same physiological processes. Based on the preceding content of this review, certain miRNAs play roles in various stages of spermatogenesis. For instance, miR-34b/c and miR-202 are involved in the entire process of spermatogenesis, including mitosis of SSCs, meiosis of spermatocytes, proliferation of SCs, and development of spermatids. Therefore, we selected miR-34b/c and miR-202 to analyze their potential synergistic effects on spermatogenesis using bioinformatics. Target genes of miR-34b/c and miR-202 were predicted using TargetScan, and their molecular functions, biological processes (**Figure 1C**), cell components (**Figure 1D**) and signalling pathways were predicted. The results indicated that miR-34b/c and miR-202 shared 126 common target genes (**Figure 1A**), including TGFBR1, TGFBR2, HGF, FGF2, HMGB2, ROCK1, USP8, and CBL. The main molecular functions of these genes were transforming growth factor-beta receptor binding, SMAD binding, and chemoattractant activity (**Figure 1B**). Several signalling pathways were involved, such as the TGF-beta signalling pathway (TGFBR2/THSD4/SP1/RPS6KB1/TGFBR1/ROCK1), adherents junction (TGFBR2/TGFBR1/ROCK1/RAP1A/PTPRF), EGFR tyrosine kinase inhibitor resistance (HGF/RPS6KB1/FGF2/PTEN), MAPK signalling pathway (TGFBR2/HGF/RASA1/MAP2K6/TGFBR1/FGF2/RAP1A), and Cushing syndrome (CREB1/USP8/SP1/RAP1A/KMT2A) (**Figure 1E**). These signalling pathways may be the primary regulatory mechanisms of spermatogenesis. In summary, bioinformatics analysis of miRNAs that co-regulate physiological processes and identification of their common upstream regulators, downstream target genes, and signalling pathways can significantly contribute to a comprehensive understanding of the role of miRNAs in spermatogenesis and male infertility.

## 5 Potential of miRNAs as biomarkers for disease diagnosis and treatment

The grading of diagnostic markers is determined by several factors, including ease of assessment, safety, rate efficiency, variability with use, and consistency across different sexes and ethnic groups. Several studies have investigated the potential use of miRNAs as useful biomarkers for the diagnosis, prognosis and treatment of diseases, including cancers, infertility, reproduction, et al. Evaluation of the expression of specific miRNAs in clinical samples may help to distinguish pathological grades, guide the treatment and predict the prognosis and treatment response.

### 5.1 Biomarkers for assisted reproductive technology (ART) outcomes.

ART success is influenced by various factors and sperm quality plays a crucial role. Preferential selection of high-quality sperm can significantly enhance early embryonic development and subsequently affect pregnancy outcomes.

In one study, the reduced expression of miR-149 in sperm was significantly associated with a high rate of good-quality embryos after 3 d of conventional *in vitro* fertilization (IVF) treatment. This suggests that decreased miR-149 expression can be used as an indicator of early embryonic development (136). Sperm DNA fragmentation (SDF) is an important indicator of sperm quality. The expression of miR-449b-5p is positively associated with SDF and the expression of miR-34c-5p. High expression of miR-34c-5p and miR-449b-5p in sperm increased the likelihood of retrieving viable embryos, suggesting that these two miRNAs are promising biomarkers for assessing sperm SDF and ART outcomes (137). Paternal miR-34c is believed to be involved in the early stages of embryonic development. Men who exhibit a positive express miR-34c are more likely to have higher rates of good-quality embryos, successful implantation, increased chances of pregnancy, and, ultimately, a higher likelihood of live birth (138). Moreover, the expression of miR-34c in sperm is closely linked to embryonic development kinetics and clinical outcomes. Consequently, miR-34c can serve as a valuable indicator of IVF outcomes, as it is beneficial for embryonic development (139). The expression of miR-34c in sperm correlates with embryonic development kinetics and clinical outcomes. Therefore, miR-34c holds promise as a potential indicator of IVF success and can provide valuable insights into embryonic development (140). A previous study demonstrated that the expression of miR-34c,-140,-21, and -375 in fresh sperm was lower in patients undergoing IVF with an average embryo score <8 than in those with an embryo score >8. Expression of the aforementioned miRNAs was positively associated with embryo score and blastomere quantity on after 3 d. The upregulation of the aforementioned miRNAs in sperm may positively regulate the development of the cleavage stage in embryos, thereby influencing the number of embryos (141). High hsa-mir-191 expression results in better early embryonic development and may improve the success rate of ART (27). The expression levels of miR-19b-3p and miR-let-7a-5p differed significantly among the spent culture media (SCM) of G1-, G2-, and G3-grade embryos. Because the expression profile of SCM is consistent with that of sperm, the expression of miR-19b-3p and miR-let-7a-5p in SCM and sperm differs significantly based on embryo quality and pregnancy outcomes. Consequently, miR-19b-3p and miR-let-7a-5p can be used as potential biomarkers to predict pregnancy outcomes (142). The expression of miR-148b-3p is low in human and mouse frozen–thawed sperm and in IVF embryos fertilized with cryopreserved sperm, leading to lower rates of embryo formation (143).

Altogether, the expression of some miRNAs (miR-149, hsa-mir-191, miR-19b-3p, miR-let-7a-5p, miR-34c, miR-140, miR-21, and miR-375) in sperm may be a useful indicator of embryo quality and pregnancy outcomes.

### 5.2 Biomarkers for varicocele grade and surgical outcome

Varicocele (Vx) is characterized by enlargement of veins in the pampiniform plexus within the spermatic cord. It is the most common treatable cause of infertility. However, distinguishing between fertile and infertile patients with Vx is challenging because of an unclear understanding of its pathogenesis. Vx negatively affects male fertility through multiple mechanisms such as scrotal hyperthermia, Leydig cell dysfunction, hypoxia, impaired testicular artery perfusion, and disruption of the BTB. In infertile men with OAT and Vx, low expression of seminal miRNA-122/-181a/-34c5. This low expression is associated with higher Vx grade and bilateral Vx and is positively correlated with sperm concentration, total sperm motility, and normal sperm morphology (66). In one study, the expression of miR-210-3p was higher in the seminal plasma of Vx patients whose semen quality became abnormal at a 2-year follow-up after surgery than in Vx patients whose semen quality remained normal. Additionally, miR-210-3p induces apoptosis in sperm cells by activating caspase-3 in patients diagnosed with Vx. Seminal plasma miR-210-3p is a promising biomarker for predicting abnormal semen quality caused by Vx (144). A study reported that the expression of miR-210-3p in the seminal plasma of men with Vx was 2.18 times higher than that in the seminal plasma of control men and significantly increased with the severity of Vx. However, the expression of miR-210-3p decreased 3 months after surgery (compared to preoperative expression), suggesting that seminal plasma miR-210-3p is a useful biomarker for screening dyszoospermia caused by Vx in clinical settings. In addition, evaluating the expression of seminal plasma miR-210-3p may guide early treatment and protect fertility (145). Another study reported that the expression of seminal exosomal miR-210-3p was significantly high in patients with grade II and III Vx but notably decreased after microsurgical varicocelectomy. miR-210-3p is negatively associated with sperm count and seminal inhibin-B. In addition, it was found to be upregulated in rats with experimental Vx (146). miR-210-3p, miR-6316, miR-190a-5p, and miR-135b-5p are involved in the regulation of the innate immune system and apoptotic signalling pathways. The expression of these miRNAs is high in Vx, suggesting that they are promising biomarkers for the diagnosing of Vx (147).

Hyperthermia and oxidative stress are the two key factors that contribute to Vx-related sperm damage. Under stress, the expression of miR-15a in sperm was significantly lower in patients with Vx than in control individuals and was negatively associated with HSPA1B. These findings provide valuable insight into Vx-related sperm damage and male infertility (148). The expression level of miR-21/-34a/-122a were significantly lower in the spermatozoa of men with VN (Vx with normal sperm) and VA (Vx with abnormal sperm) than in those of fertile men. In addition, oxidative stress levels are higher in semen samples from men with Vx than those from fertile men (149). The expression of miR−192a in the seminal plasma and testicular tissues was higher in patients with Vx without spermatozoa in the ejaculate after surgery than in patients with Vx with sperm in the ejaculate after surgery and in healthy individuals. Therefore, seminal plasma miR−192a may serve as a useful biomarker for predicting the benefits of varicocelectomy in clinical settings (56).

Altogether, the expression of seminal miR-122, miR-181a, miR-34c5, miR-210-3p, miR-6316, miR-190a-5p, miR-135b-5p, miR-21, miR-34a, miR-122a and miR−192a is related to disease severity, sperm count, and surgical outcomes in patients with Vx.

### 5.3 Biomarkers of sperm retrieval using micro-TESE in NOA

NOA is characterized by the complete absence of sperm in semen owing to impaired spermatogenesis in the testes. In some patients with NOA, testicular sperm can be retrieved using micro-TESE due to focal spermatogenesis. Subsequently, ICSI was performed to achieve successful fertilization. However, the sperm retrieval outcomes were not positive in all patients. Therefore, it is necessary to evaluate the likelihood of sperm retrieval in order to determine whether a patient is eligible for micro-TESE. In addition, selecting the optimal time for micro-TESE is a key factor in enhancing spermatogenic recovery in patients receiving certain medications. Testicular biopsies are widely performed to diagnose and predict the prognosis of NOA. However, a lack of active spermatogenic foci and severe complications are major obstacles.

In one study, four mRNAs–hsa-miR-34b-3p, hsa-miR-34c-3p, hsa-miR-3065-3p, and hsa-miR-4446-3p–were used to develop a predictive model for identifying suitable candidates for micro-TESE. The model demonstrated high predictive accuracy (AUC = 0.927) and effectively evaluated the preoperative spermatogenesis status in patients with NOA (59). The expression levels of three miRNAs (hsa-34c-5p, hsa-22-3p, and hsa-29b-3p) in seminal plasma were similar to those in testicular tissues. Additionally, these miRNAs were downregulated in USR patients compared to SSR patients. Therefore, the presence of these three miRNAs in seminal plasma may potentially serve as non-invasive biomarkers for predicting the retrieval of testicular spermatozoa before micro-TESE (41). miR-539-5p and miR-941 can serve as valuable markers for predicting the presence of spermatozoa in patients with azoospermia prior to testicular biopsy (150). Two miRNA families, miR-34b/c and miR-449, have been reported to regulate spermatogenesis in mice and are significantly downregulated in SCOS, mixed atrophy, and germ cell arrest in spermatocytes compared to normal men. Furthermore, the levels were lower in patients with USR compared to SSR patients. This suggests that these miRNAs can potentially serve as biomarkers for predicting the outcomes of micro-TESE and diagnosing male infertility (41).

In conclusion, miRNAs such as hsa-miR-34b-3p, hsa-miR-34c-3p, hsa-miR-3065-3p, hsa-miR-4446-3p, hsa-34c-5p, hsa-22-3p, hsa-29b-3p, miR-539-5p, miR-941, miR-34b/c, and miR-449 have the potential to serve as biomarkers for predicting the outcomes of micro-TESE.

### 5.4 Biomarkers for the Diagnosis of NOA

The assessment of NOA is important for andrologists and clinicians. Currently, no satisfactory diagnostic methods are available for NOA. In certain cases, a testicular biopsy is mandatory for further diagnosis. As discussed in the above sections, miRNAs in the semen and seminal plasma can be identified and used as potential non-invasive biomarkers for the diagnosis and prognosis of NOA. For example, miR-370-3p, miR-539-5p, miR-10b-3p, miR-22-5p, miR-133b, miR-210, miR-27a-3p, miR−192a, miR-19b, miR-20a-5p, miR-202-3p and miR-let-7a are upregulated and miR-34b-5p, miR-34c-5p, miR-34b-3p, miR-122, miR-122-5p, miR-31-5p, miR-516b-5p, miR-146b-5p, miR-181a, miR-374b, miR-509-5p, miR-513a-5p, miR-188-3p, miR-202-5p, miR-10b, miR-191, miR-126, miR-202-3p and miR-629-5p are downregulated in patients with NOA and may be used as potential biomarkers for the diagnosis of NOA.

Among them, the upregulation of miR-10b-3p combined with the downreguation of miR-34b-5p was used as a model to predict NOA, whose sensitivity and specificity for predicting azoospermia were 97.4% and 87.2%, respectively, with an AUC of 0.962 (40). miR-34b-5p was downregulated in NOA, and the sole target ITPR1 was upregulated; therefore, miR-34b-5p/ITPR1 was associated with the induction of apoptosis in NOA through the Ca2+ apoptosis signaling pathway, indicating that miR-34b-5p and ITPR1 may be valuable predictive biomarkers for NOA, with a specificity and sensitivity of >85% for both markers (151). Abu-Halima et al. (43) revealed that the combination of miR-34b and miR-122 with other conventional tests can improve diagnostic accuracy for detecting different forms of NOA. miR-31-5p is highly expressed in the testis and epididymis but also at lower levels in the prostate. The exosomal expression in semen samples may be useful for predicting the origin of naturally occurring azoospermia and distinguishing the types of azoospermia (54). It can also predict conserved spermatogenesis in the testes of individuals with NOA. Additionally, when combined with FSH, miR-31-5p can increase the sensitivity and specificity to 100% (150). miR-141, miR-429, and miR-7-1-3p levels were significantly higher in the seminal plasma of patients with NOA than in fertile controls. The combination of these three seminal plasma miRNAs can serve as a potential biomarker for distinguishing between NOA patients and fertile controls (39). hsa-miR-34b*, hsa-miR-34b, hsa-miR-34c-5p, and hsa-miR-122 were downregulated in the testicular tissues of patients with NOA. Receiver operating characteristic (ROC) curves were constructed to investigate the potential of these miRNAs as biomarkers for assessing male fertility. The results showed strong separation between the NOA and control male groups, and could distinguish individuals with NOA from normal control subjects with an accuracy of 99.91%, specificity of 99.69%, and sensitivity of 100% (43). miR-19b and let-7a are significantly increased in infertile males with NOA compared to fertile controls, suggesting that these miRNAs in seminal plasma may serve as potential biomarkers (61). Seminal plasma miR-192a shows promise as a potential marker for indicating the presence of spermatozoa in the ejaculate following microsurgical varicocelectomy in men with NOA and varicoceles and for pre-screening to determine which patients with NOA and varicoceles would benefit from varicocelectomy (56). Levels of miR-34c-5p, miR-122, miR-146b-5p, miR-181a, miR-374b, miR-509-5p, and miR-513a-5p were significantly lower in patients with NOA than in controls. ROC curve analyses indicated that the AUC ranged 0.822–0.921 (51). The median blood plasma levels of miR-20a-5p were higher in patients affected by NOA, positively correlated with FSH and LH, and negatively correlated with serum total testosterone and right and left testicular size, suggesting that miR-20a-5p can serve as a novel non-invasive diagnostic biomarker for male infertility (62). Hsa-miR-34c-5p expression was significantly lower in the seminal plasma of patients with NOA than in normal fertile controls. The AUCs for hsa-miR-34c-5p were 0.979 and 0.987 in the seminal plasma of patients with SA and SCOS, respectively, compared with normal fertile controls. miR-34c-5p in seminal plasma can be a potential non-invasive biomarker to diagnose patients with NOA and distinguish between different pathological types of NOA (46).

In summary, miR-10b-3p, miR-34b-5p, miR-34b, miR-122, miR-31-5p, miR-141, miR-429, miR-7-1-3p, hsa-miR-34b*, miR-19b, let-7a, miR−192a, miR-34c-5p, miR-146b-5p, miR-181a, miR-374b, miR-509-5p, miR-513a-5p, and miR-20a-5p are the most likely diagnostic markers for NOA. The detailed parameters are listed in **Table 3**.

**Table 3:** The detailed parameters of miRNA as the most likely diagnostic markers for NOA.

Ref	miRNA	comparison	position	AUC	sensitivity	specificity
(40)	miR-10b-3p	NOA=39 vs N=38	Testis	NA	76.3%	89.5%
	miR-34b-5p			NA	82.1%	76.9%
	miR-10b-3p/ miR-34b-5p			0.962	97.4%	87.2%
(150)	miR-31-5p	SA=14 vs OA=13	Exosomes in semen	0.957	92.9%	90%,
(39)	miR-141& miR-429& miR-7-1-3p	NOA=100 vs N=100	Seman plasma	0.877/ 0.832/ 0.833	87.5%/ 80.0%/ 82.5%	75.0%/ 72.5%/ 67.5%
(151)	miR-34b-5p	NOA=45 vs N=18		>0.90	>85%	>85%
(43)	miR-34b*	NOA=40 vs OA=16	Testis	0.948	99.69%	100%
	miR-34b			0.852	99.69%	100%
	miR-34c-5p			0.978	99.69%	100%
	miR-122			0.988	99.69%	100%
(61)	miR-19b	NOA=48vs N=48	Seman plasma	NA	NA	NA
	let-7a					
(56)	miR-192a	NOA=60 vs N=30	Seman plasma	NA	NA	NA
(51)	miR-34c-5p	NOA=118 vs N=168	Seman plasma	0.894	NA	NA
	miR-122			0.921	NA	NA
	miR-146b-5p			0.825	NA	NA
	miR-181a			0.875	NA	NA
	miR-374b			0.839	NA	NA
	miR-509–5p			0.822	NA	NA
	miR-513a-5p			0.825	NA	NA
(62)	miR-20a-5p	NOA=14 vs N=10	Blood plasma	NA	NA	NA
(54)	miR-31-5p	NOA=14 vs OA=13	Semen plasma	0.883	NA	NA
			extracellular vesicle	0.880	NA	NA
(46)	miR-34c-5p	SCOS=27 vs N=19	seminal plasma	0.987	NA	NA
		SA=27 vs SCOS=27		0.979	NA	NA

### 5.5 Biomarkers for other types of male infertility

KS is a prevalent sex-chromosome aneuploidy, which exhibits a diverse range of phenotypic characteristics and varying degrees of symptom severity. It is associated with symptoms such as small testicular size, hypergonadotrophic, tall stature, eunuchoid body proportions, and varying degrees of cognitive and learning difficulties. The varying phenotypes suggest the role of epigenetic mediators in the development of KS. Cimino et al. performed transcriptomic analysis of peripheral blood mononuclear cells (PBMCs) isolated from 10 patients with non-mosaic KS, 10 aged-matched healthy men and 10 aged-matched healthy women with a normal karyotype. The expression of miR-3648 and miR-3687 was found to be significantly downregulated in patients with KS, indicating that these miRNAs may be involved in the immune and metabolic disorders in male patients with KS (152). Finocchi (47) investigated the expression of miR-509-5p, miR-122-5p, miR-34b-3p and miR-34c-5p in seminal plasma samples collected from 40 patients with KS, 60 patients with NOA with a normal karyotype, 60 patients with OA and 40 healthy individuals. The expression of the four miRNAs was significantly lower in the seminal plasma of all patient groups than in that of the control group. Ibarra−Ramírez (153) conducted an analysis of miRNA expression profiles in testicular tissue samples from 4 KS patients and 5 control patients with OA using next-generation sequencing. The study revealed abnormal expression of 166 miRNAs in KS patients. A total of 7 upregulated and 20 downregulated miRNAs were subjected to separate interactome analyses, and their predicted target genes were found to be involved in spermatogenesis. Differential expression analysis of miRNAs between the PBMCs of patients with KS and healthy individuals revealed that the expression of 71 miRNAs was higher in patients with KS, that of 395 miRNAs was approximately similar in patients with KS and healthy individuals and that of 18 miRNAs was low in patients with KS (154).

## 6 Conclusion

Transcriptomic studies have identified several testis-specific miRNAs that regulate spermatogenesis and male fertility by targeting specific mRNAs. The findings of this review suggest that miRNAs expression levels are altered in patients with abnormal sperm quality and quantity. In addition, miRNAs can regulate the biological processes of undifferentiated and differentiating spermatogonia, spermatocytes, spermatids, and SCs, and serve as efficient diagnostic and prognostic biomarkers for male infertility. Collectively, the following conclusions can be drawn from this review: (1) developing a noninvasive method for diagnosing male infertility, assessing Vx grade and predicting prognosis is necessary; (2) changes in the expression of miRNAs, especially testis-specific miRNAs, may reflect various types of spermatogenic and histopathological damage in male infertility patients; (3) miRNAs are promising biomarkers for diagnosing male infertility and predicting ART outcomes. Further studies are required to elucidate the role of miRNAs in spermatogenesis at all stages of germ cell development.

## Author contributions

ZS: Writing – original draft. MY: Writing – original draft. TG: Writing – original draft. YS: Writing – original draft. ZT: Writing – original draft. XN: Writing – original draft. XC: Writing – original draft. MJ: Writing – original draft. JJ: Writing – review & editing. YL: Funding acquisition, Writing – review & editing. ML: Conceptualization, Funding acquisition, Writing – review & editing.
